# Season-dependent low basal CD86 expression promotes immune cell activation upon treatment with plasma-derived factor Ⅷ products

**DOI:** 10.1016/j.rpth.2025.103256

**Published:** 2025-11-17

**Authors:** Jessica Herzig, Josselyn Azucena Arciniega Martinez, Svenja M. Küster, Eva Ringler, Stefanie Kronhart, Gerrit J.K. Praefcke, Lilija Miller, Martina Anzaghe, Zoe Waibler

**Affiliations:** 1 Division of Immunology, Paul-Ehrlich-Institut, Langen, Germany; 2 Division of Hematology, Cell and Gene Therapy, Paul-Ehrlich-Institut, Langen, Germany

**Keywords:** dendritic cells, hemophilia A, lipopolysaccharides, risk factors, seasons

## Abstract

**Background:**

The most serious complication in treatment of hemophilia A is the development of factor (F)Ⅷ anti-drug antibodies (ADAs), while immunological danger signals seem to play a critical role in ADA development. Accordingly, we have shown in previous studies that plasma-derived (pd) FⅧ in presence of the bacterial danger signal lipopolysaccharide (LPS) synergistically activates dendritic cells (DCs), which in turn induce proliferation of (FⅧ-specific) CD4^+^ T helper cells. However, our *in vitro* assays using DC and T cells from healthy donors revealed that only cells from some donations can be synergistically activated upon treatment with pdFⅧ plus LPS, while cells from others cannot.

**Objectives and Methods:**

Therefore, we investigated a data pool of 160 donations for DC activation and 265 donations for T-cell proliferation from healthy donors collected by 3 different experimenters between 2012 and 2023 to determine parameters that correlate with pdFⅧ plus LPS-induced immune cell activation.

**Results:**

Human immune cells from healthy donors are synergistically activated by pdFⅧ plus LPS. However, neither DC activation nor T-cell proliferation depended on a specific pdFⅧ product, did not correlate with the donor’s biological sex, and was not donor- but rather time point-dependent. Analyses of different activation markers revealed seasonal differences in CD86 expression, and low expression correlated with DC activation and tumor necrosis factor-α expression.

**Conclusion:**

In fact, we could demonstrate that the meteorological season correlates with the basal CD86 expression on DC. This, in turn, determined the capability of synergistic DC activation and cytokine secretion, as well as partially impacted T-cell proliferation.

## Essentials

• Some patients with hemophilia A develop inhibitors reducing effectiveness of infused FⅧ products.
• Up to 265 donations from healthy volunteers were analyzed to define parameters correlating with immune cell activation.
• Immune activation was independent from product, sex, or donor but rather time point-dependent.
• Season-dependent CD86 basal expression on DC determines the probability of immune activation.

## Graphical abstract

**Figure:**
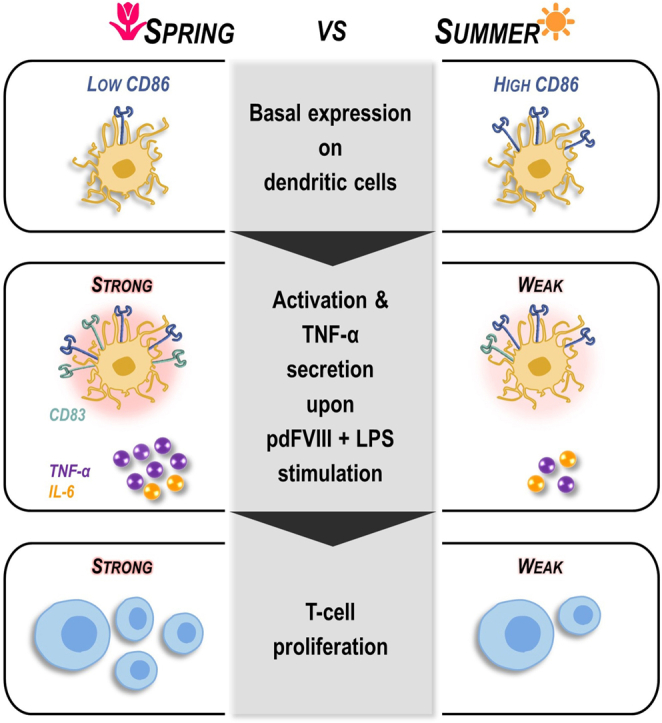

## 1 Introduction

Hemophilia A is a congenital bleeding disorder characterized by a deficiency of coagulation factor (F)Ⅷ due to mutations in the *F8* gene. As an X-linked disorder, hemophilia A predominantly affects males and manifests with prolonged bleeding episodes, both spontaneously and in response to injury or surgery [1]. Hemophilia A is classified into 3 severity stages defined by the residual activity of FⅧ in plasma resulting in different degrees of clinical manifestations [2].

Recent years have seen remarkable innovations in the treatment of hemophilia A, including the approval of a bispecific antibody and the first marketed gene therapy product [3]. However, the standard-of-care currently remains substitution therapy with 2 classes of intravenously administered biomedicines, either plasma-derived (pd) or recombinant (r) FⅧ concentrates, which aim to restore hemostasis and mitigate the risk of spontaneous bleeding events [4]. The efficacy of this therapy can be severely compromised by the development of anti-drug antibodies (ADAs), also known as inhibitors, which neutralize the infused FⅧ [5]. FⅧ inhibitors occur in about 25% to 35% of patients with severe hemophilia A [6]. While the exact mechanisms underlying inhibitor development are complex and not fully understood yet, several risk factors have been identified so far.

Genetic risk factors, in particular the severity of a patient’s specific FⅧ mutation and polymorphisms in immunoregulatory genes, eg, in the promotor region of interleukin (IL)-10 and tumor necrosis factor (TNF)-α as well as in human leukocyte antigen (HLA) class II alleles, are associated with the formation of anti-FⅧ antibodies [[7], [8], [9]]. However, inhibitors can also occur in hemophilia A patients who have minor changes in the *F8* gene. Thus, the risk of developing inhibitors cannot be explained solely by genetic predisposition [10,11]. Of note, in cases of mild or moderate hemophilia A caused by missense mutations, multiple studies have shown that immunodominant HLA-restricted FⅧ neoepitopes overlapping with the mutation site are contributing to FⅧ inhibitor development [[12], [13], [14]]. In addition to genetic risk factors, it has been discussed that treatment-related risk factors, including the number of exposure days to FⅧ, the intensity of the treatment, and the class of concentrate used, might contribute to the development of inhibitors [[15], [16], [17]]. In this context, it is still controversial whether pdFⅧ or rFⅧ products might be more immunogenic, as there are studies showing higher inhibitor rates for one or the other type [[18], [19], [20], [21]], as well as those claiming similar risks for both [15,22]. Describing the risk factors and deciphering the underlying mechanisms becomes even more complex as the development of FⅧ-specific antibodies is not limited to patients with hemophilia A. Natural FⅧ-specific autoantibodies have been found in 17% to 19% of healthy individuals [22,23]. Interestingly, previous studies have reported FⅧ-specific CD4^+^ T cells in up to 78% of healthy donors who were composed of a naïve and central memory phenotype [24,25].

Another risk factor for inhibitor development is the presence of immunological danger signals associated with events that require frequent and high-dose treatments with FⅧ, such as severe bleeding or surgery, or with infections or vaccinations [[26], [27], [28]]. These danger signals, also known as pattern-associated molecular patterns, include molecules that originate from viruses or bacteria. In this context, Kurnik et al. [28] have shown that avoiding FⅧ treatment in situations associated with high danger signal burden reduces the risk of inhibitor formation. In line with this, we have previously shown in an *in vitro* assay system that stimulation with pdFⅧ applied together with lipopolysaccharide (LPS) results in synergistic activation of human dendritic cells (DC) and T cells [[29], [30], [31]]. However, neither DC activation nor T-cell proliferation was observed in each donor/donation tested. This is in line with ADA formation observed in patients with hemophilia A, where only a proportion of patients develop ADA under not fully understood, probably multifactorial circumstances, while others do not [32]. To uncover factors which might contribute to immune cell activation upon FⅧ stimulation, we analyzed a large cohort of healthy donors, consisting of 160 donations for DC activation and 265 donations for T-cell proliferation, collected by 3 experimenters between 2012 and 2023.

## 2 Methods

### 2.1 Study design

The pseudonymized study was approved by the ethics committee of the Medical Faculty of the Goethe University Frankfurt, Germany (whole blood samples: trade number 70/15, buffy coats: waiver trade number 2023-1272). It adhered to principles of the Declaration of Helsinki (1975, revised in 2008). Each volunteer provided written informed consent. Donors, aged 18 to 65 years, included males and females.

Part of this study was conducted retrospectively using data collected by 3 experimenters >12 years (2012-2023). The data pool includes 160 donations for DC activation and 265 for T-cell proliferation from healthy donors, analyzed in 58 and 124 experiments, respectively. Blood samples originated from buffy coats (German Red Cross Blood Donor Service Baden-Württemberg Hessen, Frankfurt am Main) or freshly drawn blood. Part of the data was previously published in a different context [[29], [30], [31]].

### 2.2 DC and T-cell assay

DCs were generated from peripheral blood mononuclear cells (PBMCs) as previously described [1,2]. DCs were treated with either pdFⅧ (1 IU/mL) or LPS (0.1 μg/mL, 0.01 μg/mL, 0.001 μg/mL; *Salmonella abortus equi*; Sigma-Aldrich), or with a combination of both. Six different pdFⅧ_1-6_ concentrates (purchased commercially) were used for DC stimulation. Numeric labeling of the products corresponds to Miller et al. [29,30]. DC and T-cell proliferation assays were performed as previously described [29,30].

### 2.3 Cytokine analysis

24 hours after stimulation, supernatants were stored at −20 °C for analysis by ELISA to determine human IL-6 and TNF-α (both from R&D) according to the manufacturer.

### 2.4 Flow cytometric analysis

For flow cytometric analyses, harvested DC were stained for 20 minutes at 4 °C using monoclonal antibodies: anti-CD83 FITC (Biolegend), anti-CD86 PE (BD Biosciences), anti-CD40 Pacific Blue (Biolegend), anti-CD80 APC (EuroBioSciences GmbH or Biolegend), and anti-HLA DR APC-Cy7 (Biolegend). Harvested T cells were stained with anti-CD3 AmCyan or anti-CD4 FITC (both BD Biosciences).

### 2.5 Statistical analysis

Statistical analyses were performed using GraphPad Prism software v9.5.0. Paired comparisons used the Wilcoxon’s matched-pairs signed-rank test, while unpaired comparisons the Mann–Whitney *U*-test. Correlation analyses were done by Spearman’s correlation. The Fisher’s exact test determined significant differences in the number of synergistic activations between male and female donations. Significance levels: ∗*P* ≤ .05; ∗∗*P* ≤ .01; ∗∗∗*P* ≤ .001; ∗∗∗∗*P* ≤ .0001; not significant (ns). The correlation coefficient (*r*) was defined as: *r* < 0.3 (negligible); *r* < 0.5 low positive correlation; *r* < 0.7 moderate positive correlation; *r* < 0.9 high positive correlation; *r* > 0.9 very high positive correlation. Basal expression of samples was analyzed using FlowJo (version 10.7.1).

For synergistic DC activation analyses, mean fluorescence intensity (MFI) values for CD83 and CD86 of LPS-treated cells were set to 100% and compared with pdFⅧ plus LPS-costimulated samples. A synergism was defined as an increase in CD83 or CD86 expression of at least 10% above the LPS-induced signal [29]. For T-cell proliferation analyses, the ratio with the highest proliferation induced upon pdFⅧ plus LPS-stimulation of DC was identified per donor.

## 3 Results

### 3.1 Human immune cells are synergistically activated by pdFⅧ plus LPS

Stimulation with pdFⅧ applied along with the danger signal LPS results in a synergistic activation of human DC and T cells [[29], [30], [31]]. Nevertheless, neither DC activation nor T-cell proliferation can be observed for each donor/donation tested. In line with this, only 5% to 25% of patients with hemophilia A develop ADA upon treatment with FⅧ products [20]. To investigate which factors may contribute to these differences in synergistic DC and subsequent T-cell proliferation, a data pool of 160 (for DC activation) and 265 (for T-cell proliferation) donations from healthy subjects collected >12 years was retrospectively examined.

**Figure 1:**
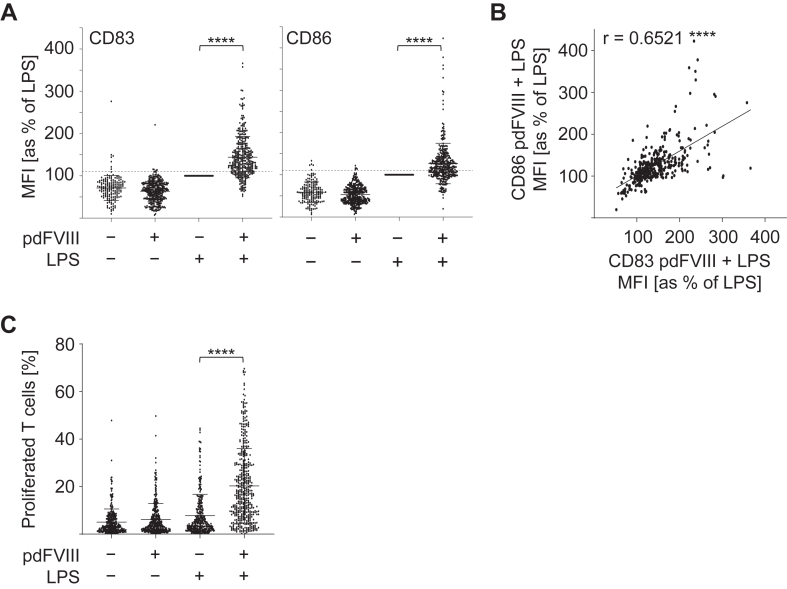
Plasma-derived FⅧ products induce synergistic activation of DC and T-cell proliferation upon costimulation with LPS. (A) Human monocyte-derived DC were stimulated with 1 of 6 pdFⅧ_1-6_ alone (1 IU/mL), LPS alone (0.001-0.1 μg/mL), or costimulated with both. Controls were left untreated. 24 hours post stimulation, DC were harvested, stained with anti-CD83 FITC or anti-CD86 PE antibodies, and analyzed by flow cytometry. For data analyses, mean fluorescence intensities of CD83 and CD86 expression were normalized to the mean fluorescence intensity (MFI) of LPS-stimulated cells (≙100%). A 10% increase in CD83 or CD86 expression over LPS alone was considered a synergistic DC activation, as previously reported by Miller et al. [29] (2015). (B) Correlation dot plot of CD83 and CD86 expression on DC upon costimulation with pdFⅧ_1-6_ plus LPS. (C) DC were stimulated as described in (A). 24 hours post stimulation, autologous PKH26- or carboxyfluorescein–succinimidyl ester-stained T cells were added and cocultured with prestimulated DC. After 9 days, T cells were harvested and stained with anti-CD3 AmCyan antibody to exclude DC. T cells were added to untreated DC as controls. Percent T-cell proliferation was analyzed by measuring the decrease in PKH26 or carboxyfluorescein–succinimidyl ester fluorescence intensity by flow cytometry. (A) and (B) In total, up to 347 single data points derived from 160 donations in 58 independent experiments were analyzed retrospectively. (C) In up to 439 data points derived from 265 donations in 122 independent experiments were examined. Statistical analyses were performed with GraphPad Prism software, version 9.5.0. Data were analyzed using the Wilcoxon signed-rank test in (A) and (C) or the Spearman’s correlation (B), respectively (∗∗∗∗*P* ≤ .0001; *r* ≥ 0.5 moderate positive correlation; mean with SD). DC, dendritic cell; F, factor; LPS, lipopolysaccharide; pd, platelet-derived.

Analyses of all donations tested from this cohort confirmed a significant synergistic DC activation upon pdFⅧ_1-6_ plus LPS-treatment as defined by an upregulation of CD83 and CD86 on DC (Figure 1A). As given in Figure 1A, the effect was more pronounced for CD83 than for CD86. However, some donations showed no synergistic activation upon costimulation. We used the same data pool to test whether the synergistic upregulation of CD83 and CD86 on DC induced by pdFⅧ_1-6_ plus LPS correlates. Indeed, we observed a moderate positive correlation of CD83 and CD86 expression when DC were pulsed with pdFⅧ_1-6_ plus LPS (Figure 1B).

As shown in Figure 1C, analyses of the 265 donations for T-cell proliferation revealed a significantly increased T-cell proliferation mediated by pdFⅧ_1,2,4,6_ plus LPS-pulsed DC when compared with each of the stimuli alone. In line with the results for DC activation and the findings obtained by Miller et al. [30], not every donation tested showed T-cell proliferation. Of note, previous studies [25,30] demonstrated that FⅧ-specific T-cell responses can be enhanced after preexpansion or repeated stimulation. In the present study, responses were assessed without prior expansion to reflect endogenous frequencies. These data demonstrate that synergistic activation of DC and T cells is not observed with every donation.

### 3.2 pdFⅧ product choice is not causative for differences in synergistic DC and T-cell activation

**Figure 2:**
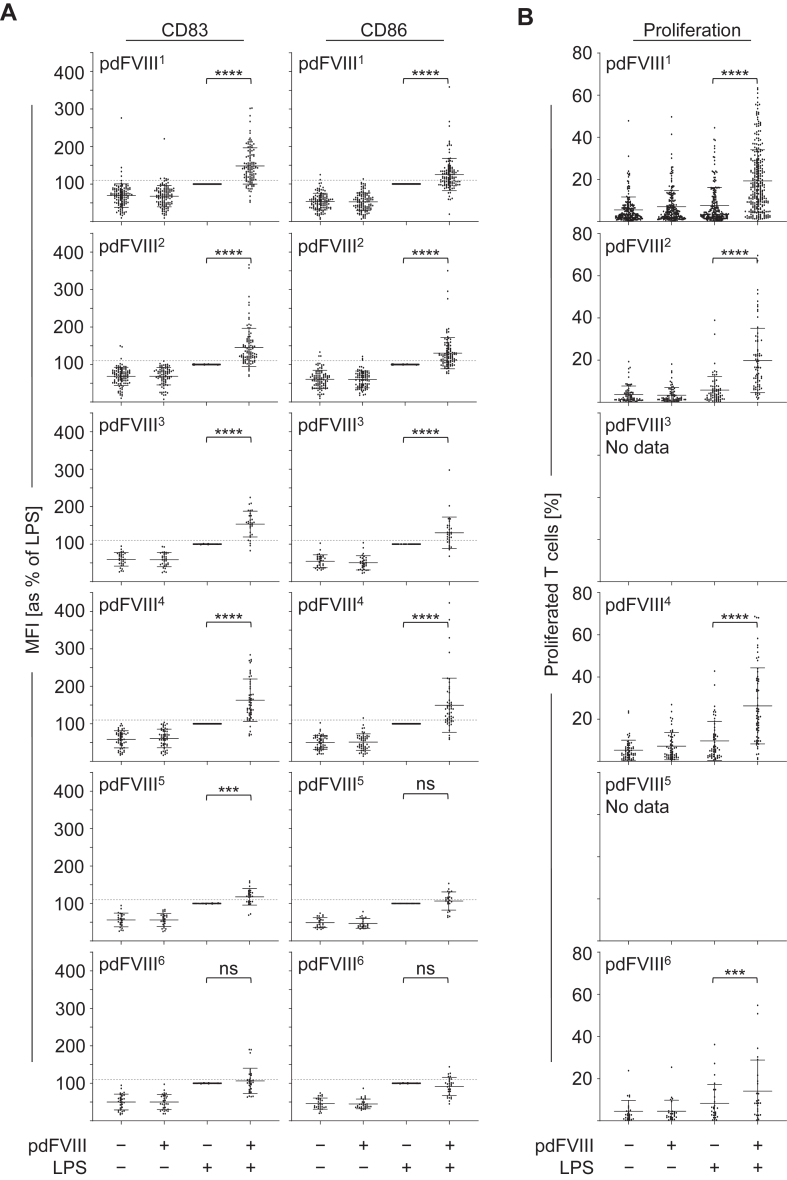
Synergistic DC activation and T-cell proliferation upon costimulation with pdFⅧ plus LPS cannot be explained by the choice of the pdFⅧ product. (A) To assess whether there are product-specific differences, data given in Figure 1A are plotted separately for all 6 pdFⅧ_1-6_ products tested. For pdFⅧ_1_, *n* = 114; pdFⅧ_2_, *n* = 97; pdFⅧ_3_, *n* = 31; pdFⅧ_4_, *n* = 51; pdFⅧ_5_, *n* = 24; and for pdFⅧ_6_, *n* = 30, donations were investigated. (B) DC were stimulated as described in Figure 1A. After 24 hours, autologous PKH26- or carboxyfluorescein–succinimidyl ester-stained T cells were added and cocultured with prestimulated DC. After 9 days of coculture, T cells were harvested and stained with anti-CD3 AmCyan antibody to exclude DC. T cells were added to untreated DC as controls. Percent T-cell proliferation was analyzed by measuring the decrease in PKH26 or carboxyfluorescein–succinimidyl ester fluorescence intensity per pdFⅧ product_1,2,4,6_ by flow cytometry. For pdFⅧ_1_, *n* = 280; pdFⅧ_2_, *n* = 74; pdFⅧ_4_, *n* = 59; and for pdFⅧ_6_, *n* = 26, data points were investigated. Statistical analyses were performed using the Wilcoxon signed-rank test with GraphPad Prism software, version 9.5.0 (∗∗∗*P* ≤ .001; ∗∗∗∗*P* ≤ .0001; mean with SD; not significant [ns]). DC, dendritic cell; F, factor; LPS, lipopolysaccharide; MFI, mean fluorescence intensity; pd, platelet-derived.

To further investigate whether the observed differences regarding DC activation or T-cell proliferation upon treatment with pdFⅧ_1-6_ plus LPS were due to product-specific differences, we examined DC activation (Figure 2A) and T-cell proliferation (Figure 2B) for each pdFⅧ product separately. As shown in Figure 2A, treatment with pdFⅧ plus LPS resulted in 9 of 12 analyses in a significant upregulation of CD83 or CD86 on DC when compared with LPS only. Notably, synergistically activated donations (≥110%) and donations below the threshold of synergistic activation (<110%) were observed for all 6 pdFⅧ products. Accordingly, some donations resulted in high T-cell proliferation upon costimulation with pdFⅧ_1_, pdFⅧ_2_, pdFⅧ_4_, or pdFⅧ_6_ plus LPS, while others did not proliferate at all. For pdFⅧ_3_ and pdFⅧ_5_, no data on T-cell proliferation were available within our data pool. Our data show that neither synergistic DC activation nor T-cell proliferation could be explained by the choice of a specific FⅧ product. Consequently, all pdFⅧ_1-6_ products were included in the following analyses.

### 3.3 Synergistic activation of DC and T cells by pdFⅧ plus LPS does not depend on the biological sex

**Figure 3:**
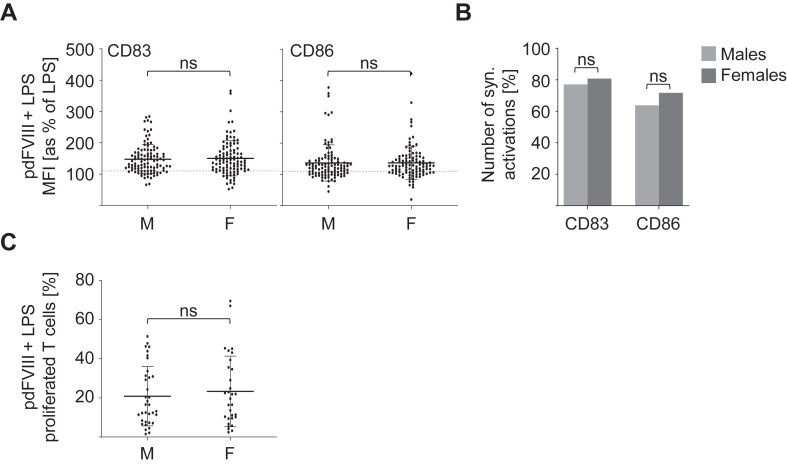
Synergistic activation of DC and T cells induced by costimulation with pdFⅧ plus LPS does not correlate with the biological sex. **(**A) and (B) DC were costimulated with 1 IU/mL pdFⅧ_1-6_ plus 0.001-0.1 ug/mL LPS. 24 hours post stimulation, DC were harvested, stained with anti-CD83 FITC or anti-CD86 PE antibodies, and analyzed by flow cytometry. For data analyses, MFI of CD83 and CD86 expression was normalized to the MFI of LPS-stimulated cells (≙100%). Donations from male (M) and female (F) healthy donors were analyzed separately. (A) The mean activation of DC induced upon costimulation with pdFⅧ plus LPS was assessed by up-regulation of the surface markers CD83 and CD86 of males and females. A 10% increase in CD83 or CD86 expression levels above LPS alone was considered a synergistic DC activation, as previously reported by Miller et al. [29] (2015). (B) The percentage of donations derived from male and female donors showing a synergistic activation (≥110%) induced upon pdFⅧ plus LPS stimulation was analyzed. (C) For comparison of T-cell proliferation between males and females, pdFⅧ plus LPS-stimulated DC were cocultured with autologous, PKH26- or carboxyfluorescein–succinimidyl ester-stained T cells for nine days. To exclude DC, T cells were harvested and stained with anti-CD3 AmCyan antibody. By measuring the decrease in PKH26 or carboxyfluorescein–succinimidyl ester fluorescence intensity, T-cell proliferation (%) was analyzed by flow cytometry. (A-C) In total, 105 or 35 data points from men and 99 or 31 data points from women were investigated for the analysis of DC activation and T-cell proliferation, respectively. Statistical analyses were performed using the Mann–Whitney *U*-test in (A) and (C) and the Fisher’s exact test in (B), both with GraphPad Prism software, version 9.5.0 (mean with SD; ns, not significant [ns]). DC, dendritic cell; F, factor; LPS, lipopolysaccharide; MFI, mean fluorescence intensity; pd, platelet-derived.

Biological sex has been shown to directly contribute to and modulate immune responses in several pathologies, such as viral infections, autoimmune or inflammatory diseases [[33], [34], [35], [36]]. Hence, we next investigated whether synergistic activation of DC and T cells might correlate with the biological sex. Therefore, all donations from our data pool were separately analyzed for male and female donors. As shown in Figure 3A, no differences in the degree of DC activation (CD83 and CD86) upon stimulation with pdFⅧ_1-6_ plus LPS were observed. In line, also the number of synergistic DC activations (in percentage) did not differ between female and male donations (Figure 3B). Consistent with the results obtained for DC activation, we found no differences in pdFⅧ_1,2,4,6_ plus LPS-induced T-cell proliferation when comparing male and female donations (Figure 3C). Collectively, these data show that synergistic activation of DC and T cells by pdFⅧ_1-6_ plus LPS does not correlate with biological sex.

### 3.4 Synergistic DC activation and T-cell proliferation triggered by stimulation with pdFⅧ plus LPS are time point-dependent

**Figure 4:**
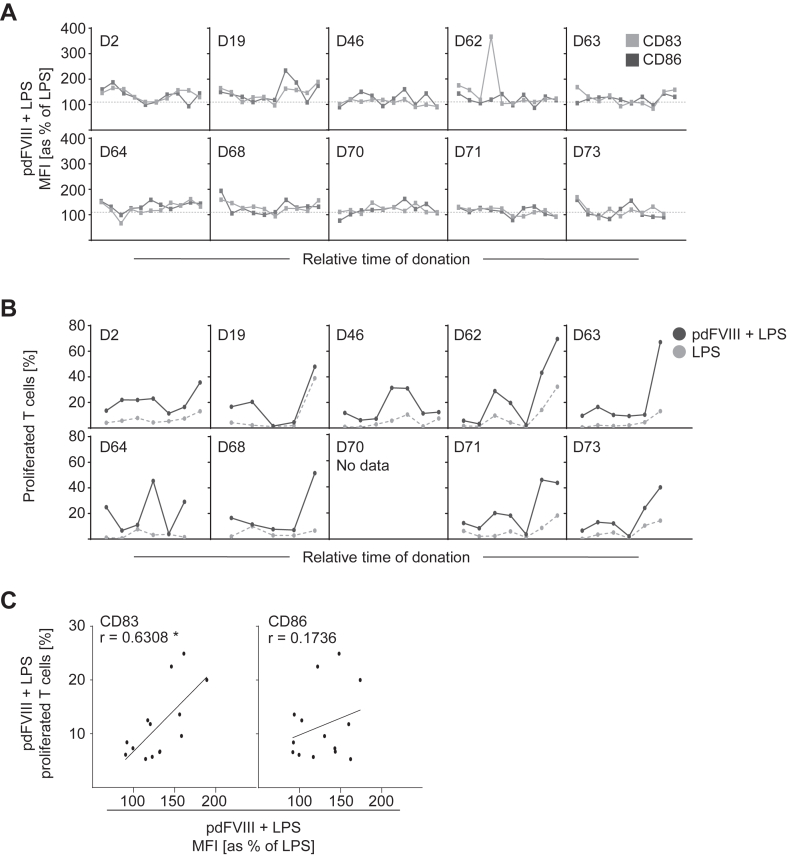
Synergistic activation of DC and T cells induced upon costimulation with pdFⅧ plus LPS is not donor-specific, but rather time point-dependent. (A) DC from a prospective group of 10 individual donors, tested at up to 10 different time points >18 months, were costimulated with pdFⅧ_2_ (1 IU/mL) plus LPS (0.001-0.01 μg/mL) for 24 hour. Untreated and LPS-treated cells served as controls (data not shown). Thereafter, DC were harvested, stained with anti-CD83 FITC and anti-CD86 PE antibodies, and their surface expression was analyzed by flow cytometry. For data analyses, MFI of CD83 and CD86 expression levels were normalized to the MFI of LPS-stimulated cells (≙100%). As previously reported by Miller et al. [29] (2015), a synergistic DC activation obtained by pdFⅧ_2_ plus LPS stimulation was defined as a 10% increase (black dashed line) in CD83 or CD86 expression compared with LPS alone. The pdFⅧ_2_ plus LPS-induced signal for CD83 is shown as light gray and for CD86 as a dark gray line for each donor. Within each donor, the tested time points for both markers are identical. Between donors, the actual time points tested may differ and are therefore shown as relative time points on the x-scale. (B) A prospective data set of 9 individual donors, tested at up to 7 different time points >5 months, was examined for T-cell proliferation induced upon costimulation (black curve) with pdFⅧ_2_ (1 IU/mL) plus LPS (0.001-0.01 μg/mL). Gray-dotted lines represent the respective T-cell proliferation induced by stimulation with LPS alone as a control for each donor at the given time point. Percent T-cell proliferation was assessed by measuring the decrease in carboxyfluorescein–succinimidyl ester fluorescence intensity using flow cytometry. (C) Nine individual donors tested up to 2 times were assessed for DC activation on day 6, as well as for T-cell proliferation after 9 days upon costimulation with pdFⅧ_2_ plus LPS. DC activation was determined by normalized MFI of CD83 or CD86, whereas T-cell proliferation (%) was determined as described above, both by flow cytometry. Data for DC activation and T-cell proliferation from the same donors at the same time points were analyzed by Spearman’s correlation (*r* ≤ 0.3, no correlation; *r* ≥ 0.5, moderate positive correlation; ∗*P* ≤ .05; not significant [ns]). Data analyses were performed using GraphPad Prism software, version 9.5.0. DC, dendritic cell; F, factor; LPS, lipopolysaccharide; MFI, mean fluorescence intensity; pd, platelet-derived.

We next aimed to investigate whether DC activation and T-cell proliferation are donor-specific. Therefore, we generated a prospective data set of 10 healthy donors who were tested 10 times >18 months. DC activation upon costimulation with pdFⅧ_2_ plus LPS was determined by analyzing upregulation of CD83 (light gray line) and CD86 (dark gray line; both Figure 4A). Interestingly, results demonstrated a rather heterogeneous pattern within a donor, showing either synergistic activation or not. Next, we analyzed T-cell proliferation donor-specifically by analyzing a prospectively generated data set of 9 healthy donors tested up to 7 times >5 months. As given in Figure 4B, the extent of T-cell proliferation mediated by pdFⅧ_2_ plus LPS-treated DC varied between time points within and between donors (black lines). In conclusion, both synergistic DC activation and T-cell proliferation are not donor-specific but depend on the time point of blood donation.

To analyze whether DC activation correlates with T-cell responses, we performed correlation analyses of our prospective data set (*n* = 13 donations, 9 individual donors; tested up to 1-2 times) [37]. Indeed, a significant moderate positive correlation of CD83 upregulation and T-cell proliferation was observed (*r* ≥ 0.5, Figure 4C). However, CD86 as DC activation marker did not correlate with T-cell proliferation in our cohort.

### 3.5 Low basal CD86 expression promotes DC activation upon treatment with pdFⅧ products

**Figure 5:**
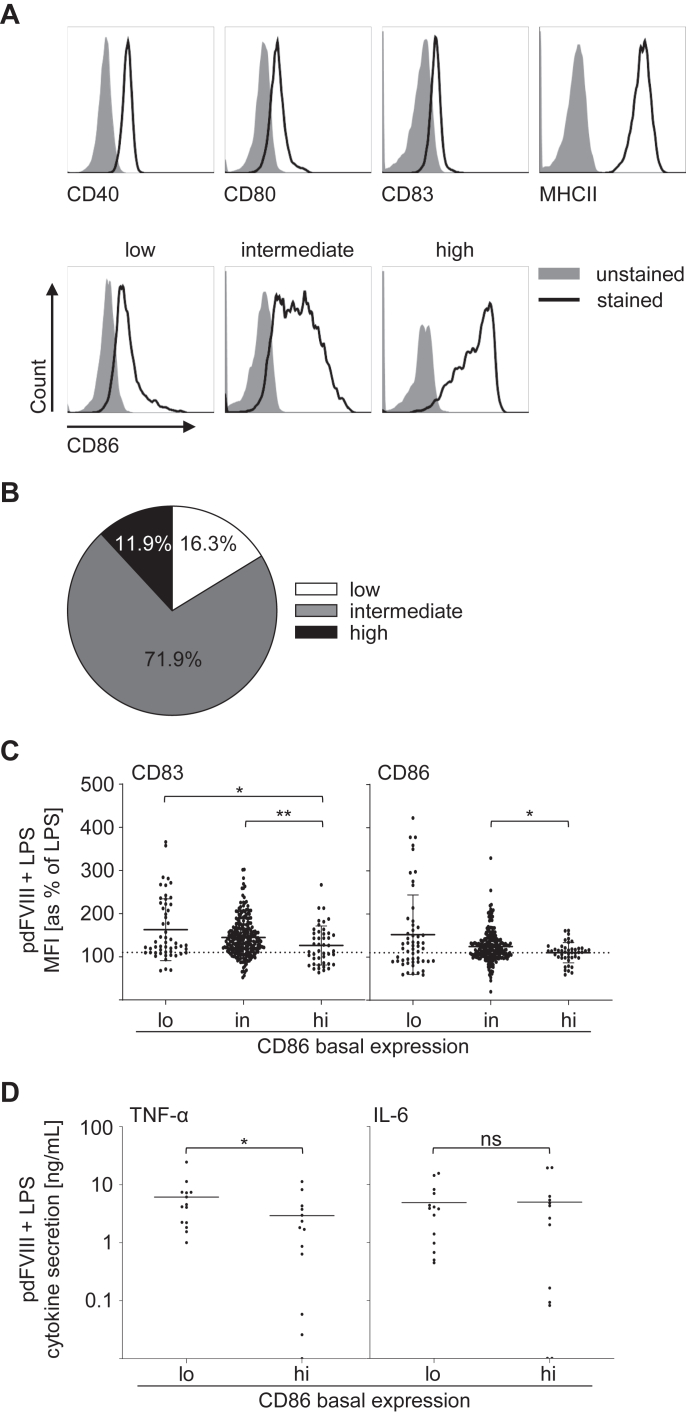
Low basal CD86 expression on DC promotes their activation upon treatment with pdFⅧ products and LPS. (A) Unstimulated DC were either left unstained (gray-shaded curve) or were stained with anti-HLA DR APC-Cy7, anti-CD40 Pac Blue, anti-CD80 APC, anti-CD83 FITC, or anti-CD86 PE antibodies (solid lines). Expression of each marker was analyzed by flow cytometry. Histograms of representative donors are shown for each surface marker. As reported in Miller et al. [29] (2015), CD86 basal expression can be classified into 3 groups: low, intermediate, and high. (B) The percentage distribution of basal CD86 expression of all 160 donations (low [white, *n* = 26]; intermediate [gray, *n* = 115]; and high [black, *n* = 19]) is given in a pie chart. (C) pdFⅧ plus LPS costimulated DC were harvested, stained with anti-CD83 FITC or anti-CD86 PE antibodies, and analyzed by flow cytometry. For statistical analyses, the mean fluorescence intensity of CD83 and CD86 expression obtained by pdFⅧ_1-6_ plus LPS costimulated DC from either low (lo), intermediate (int) or high (hi) CD86 basal expression donations were normalized to the mean fluorescence intensity of LPS-stimulated cells (≙100%). A 10% increase in CD83 or CD86 expression levels above LPS alone was considered a synergistic DC activation, as previously reported by Miller et al. (2015) [29]. (D) Supernatants of pdFⅧ plus LPS-stimulated DC from donations with either low or high CD86 basal expression were collected 24 hour poststimulation and analyzed for interleukin-6 and tumor necrosis factor-*α* (ng/mL) by an enzyme-linked immunosorbent assay (ELISA; *n* = 13-14 data points per group, performed in 3-4 independent experiments). Data were analyzed by FlowJo, version 10.7.1 and GraphPad Prism, version 9.5.0. Statistical analyses were performed with Mann–Whitney *U*-test (∗*P* ≤ .05, ∗∗*P* ≤ .01; mean with SD). DC, dendritic cell; F, factor; LPS, lipopolysaccharide; pd, platelet-derived.

As time point-dependent differences in DC activation and T-cell proliferation induced by pdFⅧ_2_ and LPS were observed, we assessed whether these differences correlate with the immune status at a given time point of blood donation. Therefore, we analyzed basal expression of activation and maturation markers CD40, CD80, CD83, CD86, or MHCII (Figure 5A; black solid line). As described previously by Miller et al. [29], 3 different levels of CD86 basal expression on DC were found: a low, an intermediate, or a high expression (Figure 5A), while expression of CD40, CD80, CD83, and MHCII was rather uniform. Therefore, we investigated all 160 donations of our data pool for their CD86 basal expression and found 12% with a low, 72% with an intermediate, and 16% with a high CD86 basal expression (Figure 5B). Notably, CD86 basal expression on DC varied over time for each donor (data not shown).

Subsequently, we analyzed whether these differences in CD86 basal expression determine DC activation upon costimulation with pdFⅧ_1-6_ plus LPS. The data summarized in Figure 5C revealed that donations with a low basal CD86 expression on DC showed the highest increase in CD83 and CD86 expression of 163% and 152%, respectively, induced by pdFⅧ_1-6_ plus LPS. In contrast, donations with a high CD86 basal expression on DC demonstrated the lowest activation of DC mediated by pdFⅧ_1-6_ plus LPS of 126% for CD83 and 110% for CD86.

Miller et al. [29] demonstrated that in addition to the upregulation of CD83 and CD86 on DC, TNF-α and IL-6 secretion is also a hallmark of pdFⅧ_1-6_ plus LPS-induced DC activation. Thus, we investigated whether secretion of TNF-α or IL-6, mediated by costimulation with pdFⅧ_1-6_ plus LPS, is determined by low or high CD86 expression on untreated DC. Consistent with the strongest upregulation of CD83 and CD86 upon pdFⅧ_1-6_ plus LPS-treatment (Figure 5B) on donations with a low CD86 basal expression, also the secretion of TNF-α, but not IL-6, was significantly higher in donations with a low CD86 basal expression on DC and vice versa (Figure 5C). In conclusion, these data indicate that low CD86 basal expression on DC promotes their activation and cytokine secretion mediated by pdFⅧ plus LPS-treatment.

### 3.6 Low basal CD86 expression in spring promotes DC activation upon treatment with pdFⅧ products

**Figure 6:**
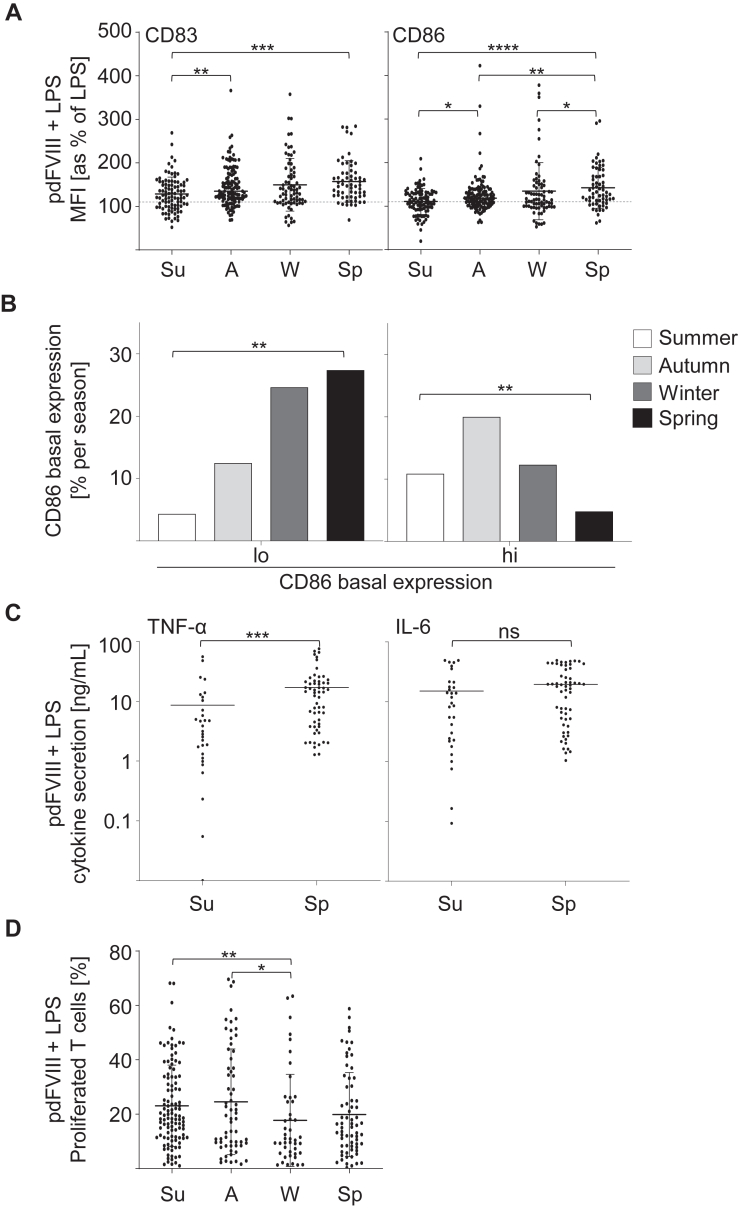
Low basal CD86 expression in spring promotes DC activation upon treatment with pdFⅧ products. **(**A) Season-wise analysis was conducted on DC co-stimulated with pdFⅧ_1-6_ (1 IU/mL) plus LPS (0.001-0.1 μg/mL) using meteorological classifications: summer (Su, June-August; *n* = 73 data points), autumn (A, September-November; *n* = 62 data points), winter (W, December-February; *n* = 92 data points), and spring (Sp, March-May; *n* = 120 data points). Untreated and LPS-treated cells served as controls (data not shown). DC activation was measured as an upregulation of DC surface markers CD83 and CD86, which were assessed by mean fluorescence intensity with flow cytometry. The pdFⅧ_2_ plus LPS-induced signal was normalized to the mean fluorescence intensity of LPS-stimulated cells (≙100%). A 10% increase in CD83 or CD86 expression levels above LPS alone was considered a synergistic DC activation, as previously reported by Miller et al. [29] (2015). (B) The distribution of low (lo) and high (hi) CD86 basal expression levels of each donation was analyzed per season (%). (C) Supernatants of pdFⅧ plus LPS-stimulated DC from donations in summer and spring were collected 24 hour poststimulation and analyzed for interleukin-6 and tumor necrosis factor-*α* (ng/mL) by ELISA (n = 30-58 data points per group, performed in 3-4 independent experiments). (D) T-cell proliferation induced upon costimulation with pdFⅧ_1-6_ (1 IU/mL) plus 0.001-0.1 μg/mL LPS of DC were analyzed per season (Su: *n* = 112; A: *n* = 64; W: *n* = 45; Sp: *n* = 68 donations). Statistical analyses were performed using the Mann–Whitney *U*-test in (A), (C) and (D) or the Fisher’s exact test in (B), both with Graph Pad Prism software, version 9.5.0 (∗*P* ≤ .05; ∗∗*P* ≤ .01; ∗∗∗*P* ≤ .001; ∗∗∗∗*P* ≤ .0001; mean with SD; not significant [ns]). DC, dendritic cell; F, factor; LPS, lipopolysaccharide; pd, platelet-derived.

It has been shown before that the season as an environmental factor influences and modulates immune responses, eg, in autoimmune diseases [[38], [39], [40]]. In order to address this, we investigated whether the season as an environmental factor could also modulate pdFⅧ_1-6_ plus LPS-induced DC activation. Hence, we grouped our data on pdFⅧ_1-6_ plus LPS-mediated DC activation into meteorological seasonal groups of summer, autumn, winter, and spring according to the time point of the respective donation. As shown in Figure 6A, the degree of DC activation as measured by upregulation of CD83 and CD86 was significantly higher for donations obtained in spring when compared with donations in summer. Thus, meteorological season indeed correlates with the degree of pdFⅧ_1-6_ plus LPS-mediated DC activation. Consequently, we aimed to identify whether there is a relationship between season and the observed CD86 basal expression. Therefore, we analyzed the frequency of low or high CD86 basal expression of all 160 donations within our data pool season-wise (Figure 6B). Indeed, the highest number of donations with a low CD86 expression, along with the lowest number of high CD86 expression, was observed in spring. In line with that, significantly higher secretion of TNF-α (17 ng/mL), but not IL-6, was observed in spring when compared with summer (Figure 6C).

Finally, we investigated whether season might contribute to differences in the degree of T-cell proliferation mediated by pdFⅧ plus LPS as well. We analyzed all 265 donations within our data pool for T-cell proliferation per meteorologically defined seasons. However, in contrast to seasonal results on DC activation, we found the highest T-cell proliferation rate in autumn and the lowest in winter (Figure 6D).

## 4 Discussion

The immunological mechanisms underlying the development of FⅧ inhibitors in patients with hemophilia A and the potential risk factors are not yet well understood. In this context, we have previously shown in 2 assay systems with healthy donors that the treatment of DC with pdFⅧ plus LPS results in synergistic DC activation, which in turn induces the proliferation of autologous T cells when compared with each of these substances alone. However, not all donations examined in these studies showed DC or T-cell activation upon treatment [[29], [30], [31]]. In order to investigate factors that may contribute to pdFⅧ plus LPS-induced immune cell activation and potentially elucidate the observed differences between donations, we investigated a large data pool consisting of 160 donations for DC activation and 265 donations for T-cell proliferation.

The individual’s sex as a biological variable has become an emerging parameter to study in the last 30 to 40 years, as it is now described to affect several functions of the immune system by modulating both innate and adaptive immune responses across diverse species [36]. However, in our experimental setting, we did not find a correlation between the biological sex of a donor and the synergistic activation of DC and T cells mediated by pdFⅧ plus LPS. Interestingly, Hu et al. [24] investigated differences in the proliferative response of CD4^+^ T cells from healthy donors upon stimulation with FⅧ or FⅧ peptide pools. They reported that the FⅧ protein induced a significant higher proliferative response of CD4^+^ T cells in males (*n* = 62) than in females (*n* = 28), whereas proliferation of CD4^+^ T cells upon stimulation with 20 amino acid FⅧ peptide pools was comparable in male and female donations. Conflicting results might be explained by the rather small sample size of female donations in the cited study. However, it should be noted that hemophilia A predominantly affects males, although females may also be affected in certain cases [41]. This, combined with our findings that synergistic activation of DC and T cells did not correlate with biological sex (Figure 3), confirmed our decision to include female donors in all of our analyses.

Until recently, CD86 was primarily known as a costimulatory molecule that is constitutively expressed at minimal levels on DC and upregulated upon activation [42]. CD86 interacts with the CD28 coreceptor on T cells, delivering a costimulatory signal essential for T-cell activation, particularly in T-cell dependent immune responses, such as in ADA development [43].

Interestingly, we observed that the season as an environmental factor affects the basal CD86 expression on DC, which subsequently determines the potential and degree of DC activation (surface marker upregulation and cytokine secretion) and, to some extent, T-cell proliferation. We found that unstimulated DC can exhibit varying levels of basal CD86 expression (low, intermediate, or high). In line with our previous findings, CD86 expression levels determined the extent of synergistic DC activation and TNF-α secretion, upon stimulation with pdFⅧ plus LPS [29]. Along this line, results of a study by Radvanyi et al. [44] showed that DC can exhibit a low to intermediate CD86 basal expression that can transition into a high CD86 state following low-dose stimulation with interferon α.

To this end, we could show in a former study that blocking of CD86 and MHCII significantly reduced T-cell response in the context of FⅧ, pinpointing to a crucial role of CD86 in T-cell mediated responses [30]. Moreover, it is known that DC can downregulate CD86 expression following exposure to the anti-inflammatory cytokine IL-10 [[45], [46], [47]].

However, to our knowledge, varying basal levels of CD86 expression on unstimulated DC that influence their activation potential have been scarcely studied and reported. Therefore, little is known about parameters that might be modulators of CD86 in healthy subjects. In general, environmental influences such as seasonality have been shown to affect immune cell frequencies and cytokine production in healthy individuals [38]. In addition, UV exposure was shown to alter DC maturation, downregulate costimulatory molecules including CD86, and induce tolerogenic DC phenotypes [48,49]. Likewise, pollen-derived bioactive lipids were shown to modulate DC function by decreasing IL-12 production of DC and thereby shifting T-cell responses toward a Th_2_ phenotype [50]. While systematic studies of basal CD86 expression across seasons are lacking, these findings might provide a plausible framework to interpret the intra- and interindividual variability observed in our study.

Our seasonal analyses revealed that the highest DC activation upon stimulation with pdFⅧ plus LPS occurred in spring (150%), which coincided with the highest number of donations exhibiting low CD86 basal expression levels and a higher secretion of TNF-α in spring when compared with summer. Seasonal variations have been shown to affect immune responses in other contexts, such as infectious and autoimmune diseases, which might also impact ADA development [39,40,51]. For instance, composition of T-cell subsets and cytokine secretion by immune cells have been reported to fluctuate with seasonal changes, potentially due to variations in vitamin D levels, sunlight exposure, infections, and pollen exposure [38,52,53]. In this context, Horst et al. [38] revealed that markers of inflammation measured in plasma, such as IL-18, along with cytokine production capacity of PBMC *in vitro*, particularly upon stimulation with influenza virus, displayed seasonal variability, with peaks observed in winter. Moreover, they investigated seasonal changes in various immune cell subset counts and found CD4^+^ and CD8^+^ T cells to be influenced by seasonal shifts. Notably, CD4^+^CD25_high_ regulatory T cells were more abundant in autumn when compared with the other seasons [38]. In our study, CD86 basal expression on DC and their capability to be synergistically activated correlated with the season. However, T-cell proliferation was not in line with these findings. It should be noted that we analyzed the proliferation of all CD4^+^ T cells, regardless of their subtype. Whether results differ for individual T-cell subsets, which can vary depending on the season as described above, cannot be retrospectively analyzed based on our data set.

As abovementioned, vitamin D is a factor described to modulate immune responses and is connected to seasonal variability. It is well-known that levels of the active form, vitamin D3, fluctuate in the body primarily due to variations in UVB radiation from sunlight, which changes with the time of year and geographic location/latitude. In this context, a study by Hays et al. [54] involving a retrospective analysis of 1256 adult patients >20 years, demonstrated significant seasonal fluctuations in vitamin D3 levels. They identified the highest vitamin D3 levels during summer and the lowest during spring and winter. To assess the effect of vitamin D3 concentration regarding immune cell function, recent research by Vanherwegen et al. [55] demonstrated that sufficient levels of vitamin D3 promote the induction of a tolerogenic DC phenotype. In turn, these tolerogenic DC induce the activation of regulatory T cells, which help to suppress autoimmune reactions [55]. Furthermore, Khoo et al. [56] investigated effects of vitamin D3 on cytokine production by human PBMCs. They found that pre-incubation with vitamin D3 significantly reduced the secretion of TNF-α as well as IL-6 in PBMCs that were stimulated with the toll-like receptor ligands LPS or Pam3Cys. In line with this, they also observed that naturally occurring vitamin D3 levels were highest in summer, correlating with a decreased secretion of cytokines, such as TNF-α, upon stimulation with LPS or Pam3Cys, respectively [56].

The present study was conducted with cells from healthy donors. While this does not directly address inhibitor development in patients with hemophilia A, access to sufficient patient samples is limited and often technically challenging. Nevertheless, FⅧ-specific immune responses are also detectable in the healthy population. Natural FⅧ-specific autoantibodies have been found in 17% to 19% of healthy individuals [22,23] and FⅧ-specific CD4^+^ T cells have been reported in up to 78% of healthy donors, displaying both naïve and central memory phenotypes [24,25]. Together, these findings support the relevance of data derived from healthy donors for elucidating fundamental mechanisms of FⅧ immunogenicity [[29], [30], [31]]. Taken together, our results propose that ADA formation upon pdFⅧ treatment is a complex, multifactorial process. Notably, the time point of blood donation, particularly the meteorological season, impacts the immune status of DC, as reflected by basal CD86 expression. This baseline activity shapes the DC’s capacity for synergistic activation (via surface marker regulation and cytokine secretion) and, to some extent, T-cell proliferation. However, whether the observed seasonal effect on DC activation by pdFⅧ plus LPS and induced T-cell proliferation translates similarly into season-dependent ADA development in patients with hemophilia A will be a matter of future investigations. Given the potential role of vitamin D3 as a modulator of pdFⅧ plus LPS-induced immune responses, assessing vitamin D3 levels in healthy donors and patients with hemophilia A, as well as their supplementation behavior, could yield valuable insights. In addition, strategies to modulate the basal expression of CD86 should be investigated as this may provide a way to reduce immunogenicity. The findings obtained in this study contribute to the understanding of selecting the correct timing for FⅧ substitution therapy and may improve personalization of FⅧ therapy, ultimately supporting more tailored and effective treatment approaches for patients with hemophilia A.
